# Outbreak of epidemic keratoconjunctivitis caused by adenovirus in medical residents

**Published:** 2009-03-20

**Authors:** Carlos Pantoja Melendez, Margarita Matias Florentino, Irma Lopez Martinez, Herlinda Mejia Lopez

**Affiliations:** 1 Institute of Ophthalmology “Conde de Valenciana” Research Unite, Mexico City, Mexico; 2 Institute for Diagnosis and Epidemiological Reference, Mexico City, Mexico

## Abstract

**Purpose:**

The present work documents an outbreak of epidemic keratoconjunctivitis among ophthalmology residents, its influence in the presentation of the community cases, the use of molecular techniques for its diagnosis, and the implementation of successful control measures for its containment.

**Methods:**

Isolation of the etiologic agent was achieved using cultured African green monkey kidney epithelial cells (VERO). Through molecular tests, such as polymerase chain reaction (PCR) and DNA sequencing, the genotype of the isolated virus was identified. The sequences obtained were aligned with data reported in the NCBI GenBank. A scheme of outbreak control measures was designed to enforce correct sanitary measures in the clinic. The statistical program, Epi info 2002, and openepi were used to determine the attack rate. The Excel Microsoft® program was used to elaborate the endemic channel.

**Results:**

Nine of the ten samples studied were isolated from the culture and identified by Adenovirus-specifc PCR. Sequencing allowed identification of Ad8 as the agent responsible for the outbreak. The attack rate was 24.39 cases per 100. The epidemic curve allowed identification of a disseminated source in the Institute of Ophthalmology “Conde de Valenciana.” It was not possible to calculate the incubation periods among the cases. The endemic channel showed the presence of an epidemic keratoconjunctivitis among the patients that had been cared for at the out-patient services of the institute.

**Conclusions:**

One outbreak of a disseminated source caused by Ad8 was detected in the institute among its medical residents, probably associated with relaxation of the habitual sanitary measures during an epidemic of hemorrhagic conjunctivitis among the patients cared for at the institute. The proposed scheme to control the outbreak allowed for its containment and controlled the epidemic of associated cases.

## Introduction

The interaction between clinicians and patients produces a dynamic that can affect both and forms a part of the risk associated with medical practice. The clinician can sometimes become the main agent for the spread of disease by omissions, which usually do not affect the medical personnel or the patients. However, under certain circumstances, members of the medical workforce can become risks for their own health and that of their patients by becoming a vector for disease transmission. In this study, we report on an outbreak of conjunctivitis involving both clinicians and patients.

Human adenovirus (Ad) belongs to a family comprising about 100 members that are grouped into six subgenera (A-F) based on several biochemical and molecular criteria. Fifty-one Ad types have been identified as human pathogens, some of which are able to survive under adverse conditions outside of the human body [1,2]. A typical Ad infection includes a 5–12 day incubation period before the onset of symptoms followed by a 14 day transmissibility period.

The various Ad types have different tissue tropisms with observed signs and symptoms typically involving the visual, respiratory, or gastrointestinal systems. Subgenus C Ads are predominantly responsible for respiratory infections but can also cause acute follicular conjunctivitis either as part of a respiratory-pharyngeal syndrome or as a separate disease entity. These diseases are often accompanied by significant preauricular lymphadenopathy. Subgenera B and E (Ad3, Ad7, and Ad4) can also be associated with this syndrome [1,3].

Keratoconjunctivitis usually starts as an acute follicular conjunctivitis affecting both eyes with subsequent injury appearing in more than 50% of cases. Lesions are characterized by diffuse, point-like epithelial keratitis that can heal or else can evolve into subepithelial injuries that last for varying periods of time even months and that produce visual alterations affecting the patient’s quality of life. Some members of subgenus D, particularly Ad8, Ad19, and Ad37, can cause epidemic outbreaks with several of the aforementioned signs and symptoms being present. Genotype variants of Ad4, have been associated with outbreaks of conjunctivitis [3-9].

Ad transmission can occur by several means including direct contact, fecal-oral transmission, and contact with non-chlorinated or insufficiently chlorinated water. Some Ad types can establish persistent and non-symptomatic infections while others can cause seasonal sporadic infections such as that occurring with the keratoconjunctivitis epidemic. Most of the described epidemic outbreaks represent infections of a common source, which can include inadequately chlorinated swimming pools (not always confirmed) or contaminated ophthalmology units. The occurrence of secondary infections is common among families, friends, and fellow workers through either direct or indirect transmission. Transmission occurs by several pathways including direct contact through ocular secretions of an infected person or through contact with instruments, solutions, or objects that have been indirectly contaminated. Direct contact between clinical personnel and their patients plays a fundamental role in nosocomial outbreaks [1,3]. It is also worth mentioning that since adenoviruses can be excreted for long periods of time after infection, their presence is not necessarily associated with ongoing disease [3].

Adenoviruses can be identified by virus isolation in sensitive cell cultures, although this is time consuming. Molecular methods such as restriction fragment length polymorphisms (RFLPs) and polymerase chain reactions (PCR) also provide useful and sensitive analytical tests for the rapid diagnosis of Ad [8,10,11]. Differential diagnosis of keratoconjunctivitis must be made regarding the pharyngoconjunctival fever produced by Ad3 or Ad7, which appears in children and young adults, as well as from hemorrhagic conjunctivitis caused by an enterovirus and conjunctivitis induced by chlamydia [9,10,12].

In this report, we describe an outbreak of epidemic keratoconjunctivitis occurring among medical residents of an ophthalmology facility in Mexico City, focusing on its influence on the presentation of communitarian cases (secondary), its diagnosis through molecular techniques, and the measures taken for its containment.

## Methods

A retrospective interventional study, known as an outbreak study, was performed.

### Outbreak identification

An outbreak of conjunctivitis was documented in the Institute of Ophthalmology “Conde de Valenciana”. Several cases of conjunctivitis with similar clinical characteristics were identified among medical residents between February and October of 2006.

### Operational case definition

Suspicious cases of keratoconjunctivitis during 2006 in the Institute of Ophthalmology “Conde de Valenciana” among the medical personnel, and people whom there are been in contact with the medical residents.

### Index case definition

The initial patient in the cohort was identified as being diseased by an epidemiological investigator.

### Clinical symptoms

Signs and symptoms were compatible with a probable viral conjunctivitis. These included tears, foreign body sensation, palpebral edema and hemorrhage, pain, photophobia, blurred vision, and serous secretion (Table 1). All medical residents with follicular conjunctivitis and one or more of the described symptoms were referred to the clinical laboratory for identification of the responsible agent.

**Table 1:** Clinical manifestations.

**Signals or symptoms**	**Number of cases**	**Average cases (%)**
Tearing	10	100
Conjunctival inflammation	10	100
Foreign body sensation	9	90
Palpebral edema	9	90
Pain	8	80
Photophobia	8	80
Blurred vision	7	70
Conjunctival hemorrhage	5	50
Serous secretion	5	50
Keratitis	5	50
Periorbital edema	5	50
Preauricular lymphadenopathies	5	50
Subepithelial infiltrates	5	50
Ptosis	4	40
No secretion	3	30
Purulent secretion	2	20

The signs and symptoms of keratoconjunctivitis are observed.

### Virus isolation

Conjunctival scrapings were taken from 10 medical residents presenting with keratoconjunctivitis. Samples were collected with calcium alginate swabs and stored in Hank’s buffer supplemented with 50 mg/ml gentamicin, 500 U/ml penicillin-streptomycin, 1 mg/ml Amphotericin B, and 5% serum albumin (Sigma Chemical, St Louis, MO). Each sample was inoculated into cultured African green monkey kidney epithelial cells (VERO; ATTC, CCL-81; Global Bioresource Center, Rockville, MD). Ad cell cultures with cytopathic effect were assayed by direct immunofluorescence (DI) with an Adenovirus DFA kit (Chemicon Inc. International, Temecula, CA), using propidium iodide as the contrast dye [13]. All DI positive cell cultures were then subjected to Adenovirus-specific PCR.

### Viral DNA extraction

Supernatants were collected from PCR positive cell cultures and frozen–thawed three times before DNA extraction to obtain a larger viral DNA yield. DNA isolation was performed with a QIAamp mini-kit as instructed by the manufacturer (Qiagen Sciences, Germantown, MD). DNA isolates were stored at −20 °C until further processing.

### Adenovirus-specific polymerase chain reaction

To demonstrate the presence of Ad, PCR was performed using the modified primers ADRJC1 5′-GAC ATG ACT TTC GAG GTC GAT CCC ATG GA-3′ and ADRJC2 5′-CCG GCT GAG AAG GGT GTG CGC AGG TA-3 '. This yielded a 140 bp product corresponding to the highly conserved DNA region coding for the carboxyl end of the monomeric protein II that forms the trimeric pseudohexagonal base of the adenovirus hexon [4]. Reaction conditions were the same as those reported by Elnifro et al. [14]. The PCR products were analyzed in 1.5% agarose gels staining with ethidium bromide (1 μg/ml; Sigma). A Ready-Load 100 bp DNA Ladder was used as a standard marker for molecular weight (Invitrogen Co L.T., Carlsbad, CA).

### Adenovirus tipping

The 140 bp PCR products were purified with QUIAEX II columns following the manufacturer’s instructions (Qiagen Sciences). Purified PCR products were sequenced with the ADRJC1 primer using the BigDye Terminator kit (Applied Biosystems, Foster City, CA). The DNA sequences obtained were compared to sequences reported in GenBank with the BLAST algorithm. Alignment was made using the MULTALIN algorithm [15].

### Outbreak analysis

Statistical analysis was conducted with Epi Info 2002 and openepi (United States Department of Health and Human Services, Centers for Disease Control and Prevention, 1600 Clifton Rd. Atlanta, GA) to determine the attack rate. Microsoft® Excel (Microsoft Corporation, Redmond, WA) was used to elaborate the endemic channel.

## Results

**Figure 1:**
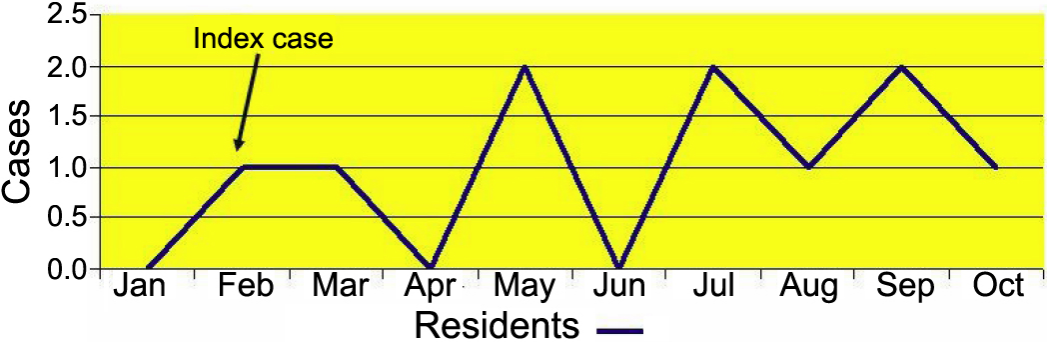
Epidemic curve. It is observed that the cases of keratoconjunctivitis appeared in a dispersed way in the study period that is compatible with the epidemiological term “scattered source,” characterized by the present sporadic cases epidemiologically related.

Ten medical residents with conjunctivitis were identified. Their average age was 27.7 years; eight were female and two were male. None of the residents who were affected by the hemorrhagic conjunctivitis indicated that any cases of conjunctivitis had occurred among his or her relatives. To characterize the outbreak, an epidemic curve was constructed. Figure 1 shows the extended duration for the appearance of cases as well as the classic image of a disseminated source of infection. An attack rate of 24.39 cases per 100 residents was estimated.

### Outbreak control

**Figure 2:**
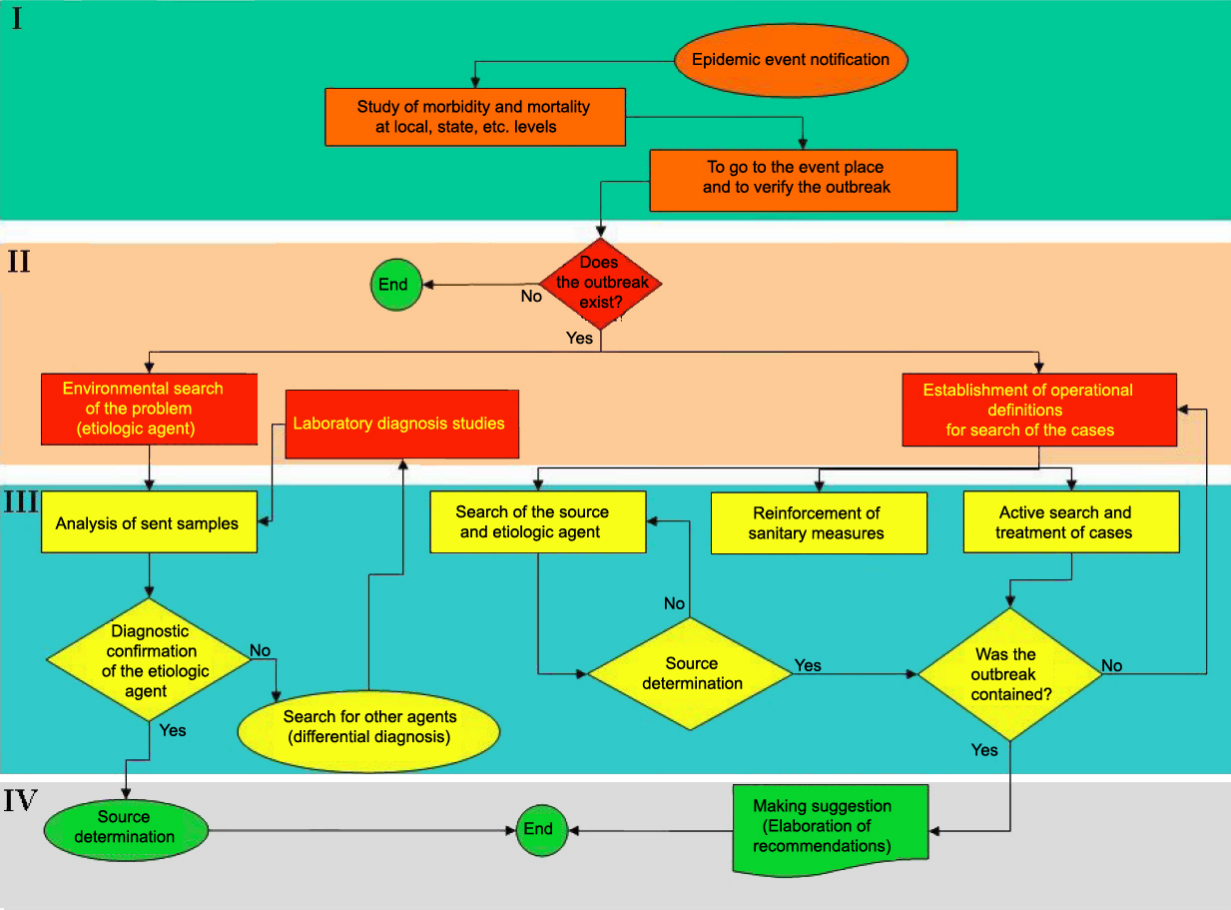
The four basic phases of attention to an outbreak. I) Knowledge of the problem (characterized by the presence of unexpected cases epidemiologically related; II) decision making (the cases not waited for are documented and the measures of search, diagnosis, attention of cases, and measures of control of place where they appeared are decided on; III) attention to the outbreak (clinical and epidemiological methods are applied to the patients and the source of the outbreak is determined, the agent (if it is feasible), and the sanitary measures for controlling the outbreak are applied; and IV) resolution (In the last stage are determined the causes and recommendations are released to avoid the presentation of new cases and knowledge is generated for the avoidance of future outbreaks.

Outbreak control measures were undertaken to break the transmission chain and to correct the deviation from habitual sanitary measures that had occurred as described in Figure 2. In phase I, the existence of the event becomes evident through notification or as part of the habitual epidemiological monitoring system. Phase II identifies modifications in the expected morbidity. In this phase, guidelines are established for the search of the causal agent, and the transmission chains are determined (if they exist). In phase III, the activities for the containment of the outbreak are implemented (directed at attending to the cases and interrupting the transmission chains). In phase IV, the sanitary recommendations and sanitation proposals are elaborated following the determination of the source to avoid occurrence of new outbreaks.

The following recommended control measures were adopted:

1. On October 19, 2006, all of the medical personnel in the institute were alerted as to the occurrence of the hemorrhagic keratoconjunctivitis outbreak with the etiologic agent being clinically diagnosed and confirmed by the laboratory as Ad8 .
2. All residents with symptoms were declared sick for at least 15 natural days.
3. Hand washing before and after attending each patient was strictly enforced.
4. Disposal of all remnant solutions and eye drops used by patients was made mandatory.
5. Shared use of solutions and eye drops was strictly prohibited.
6. Exhaustive sanitation of the outpatient service areas was enforced.
7. Hygienic measures in the outpatient service areas were reinforced.
8. A campaign was initiated to inform personnel working at the institute about the outbreak occurrence and the containment and control measures put into place.
9. A campaign was initiated to communicate information and to enhance awareness among the patients attending the institute on hygienic behaviors that would contribute to containment and control of the outbreak.

All cases of epidemic keratoconjunctivitis were investigated among the ophthalmology residents in all areas of the institute where cases were identified. A retrospective and active search of the cases was made. Data including name, age, gender, home address, year of residency, familial cases, onset of symptoms, clinical characteristics, antecedents, and sequels were collected and analyzed.

### Associated cases

**Figure 3:**
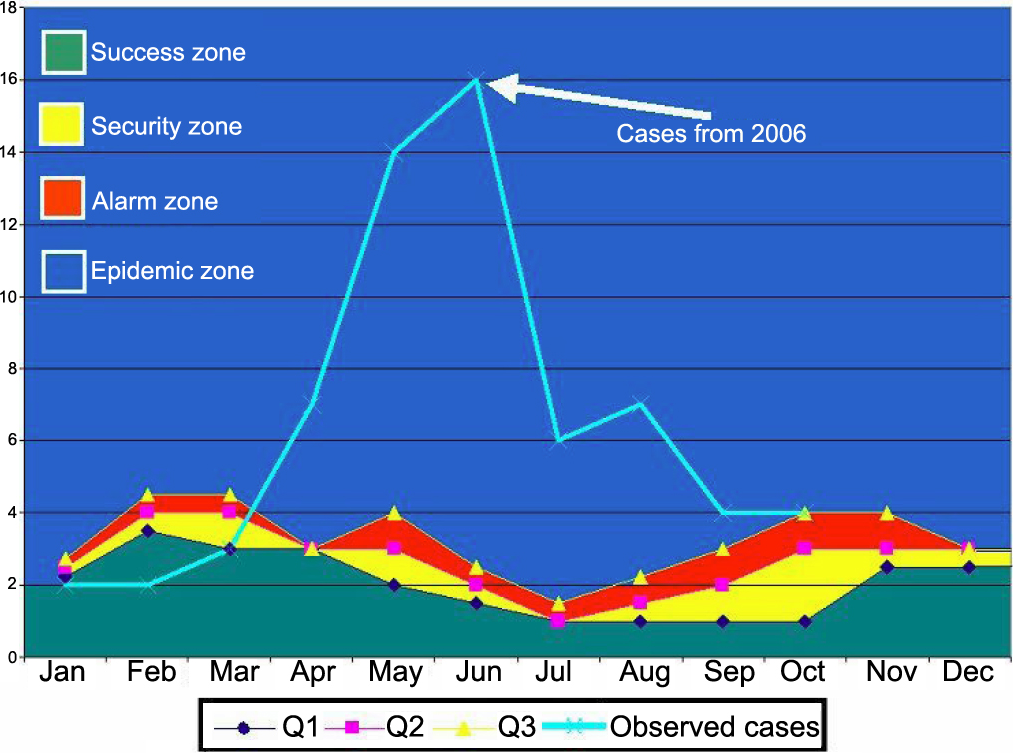
Endemic channel. Cases observed in 2006 lie within the epidemic zone after the appearance of the index case. The zones of success, security, and alarm determine the habitual behavior of the cases of keratoconjunctivitis, in contrast, the cases in 2006 were in compatible parameters with an epidemic.

To determine whether the incidence of cases in the hospital personnel had an impact on the patients attending consultation, an endemic channel with data from 2001 to 2005 was constructed and compared to the observed cases in the year 2006. The behavior of the cases in the outpatient service determined the source. Figure 3 reveals an increase in the observed cases with regard to the expected cases in the population attending the outpatient service (cases below the epidemic zone). This increment appeared at the beginning of March at the beginning of the epidemic zone after the index case among the medical residents (first case of the outbreak) was identified. There was a significant increase in the observed cases with respect to those expected.

It was not possible to calculate incubation periods for the cases that appeared in the hospital because the source was disseminated.

### Isolation and molecular tests

**Figure 4:**
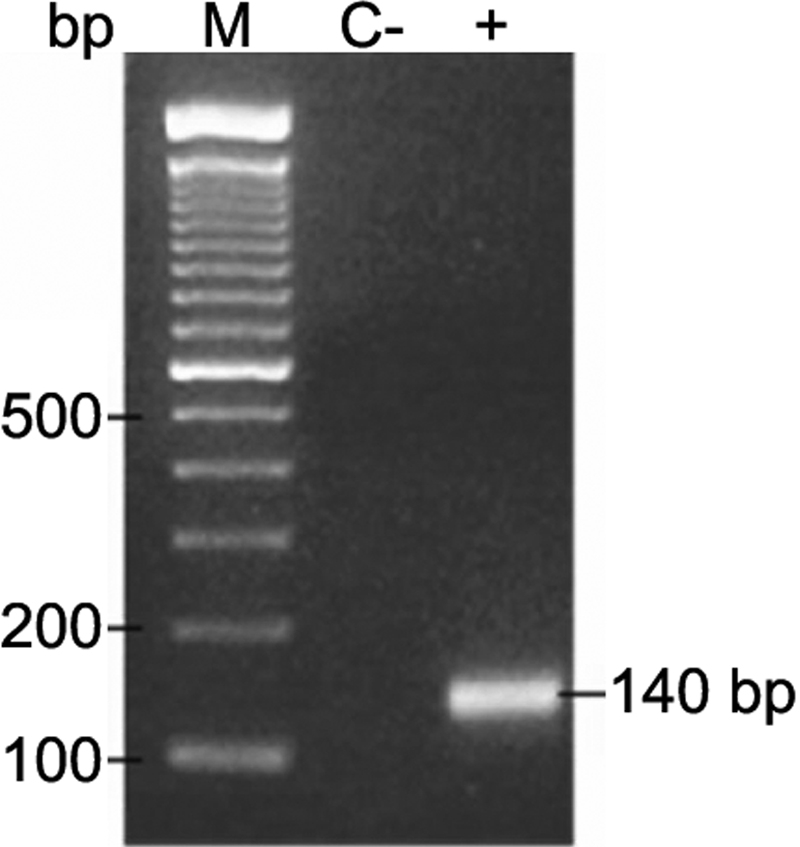
1.5% agarose gel showing a representative PCR sample. This 140 pb product corresponds to the highly conserved DNA region coding protein hexon, common to all adenovirus. Line 2, negative control; Line 3, 140 bp amplification; M, standard marker, Ready-Load 100 bp DNA.

The etiologic agent was isolated from 9 of 10 samples collected from the medical residents. The 10 studied samples were positive for Adenovirus-specific PCR products. Figure 4 shows the 140 bp amplification product representative of one positive result.

### Typing of the adenovirus

The etiologic agent was identified by sequencing. The nucleotide sequences obtained in all cases had 98% homology to nucleotide sequence accession number DQ149614 at GenBank.

## Discussion

Epidemic keratoconjunctivitis outbreaks involving adenoviruses have been reported in several ophthalmology clinics [16-18]. The disseminated outbreak that occurred in our institute took place among the medical residents. The conjunction of variables of time, place, and persons indicated that it was a hemorrhagic conjunctivitis outbreak.

The epidemic curve indicated that the transmission of the outbreak was a scattered type because all of the cases followed one another sequentially over a prolonged duration rather than with a sudden onset, which would indicate a common source. An investigation of all of the areas in which cases of conjunctivitis had appeared indicated that the contributing factors to dissemination had been a lack of hand washing, a lack of physical barriers (gloves and/or lenses) while attending to patients, and inadequate disinfection of the medical equipment. This was demonstrated by the successful implementation of the actions mentioned in the flowchart directed to the reestablishment of sanitary control measures (Figure 2).

The endemic channel (Figure 3) allowed the determination of an epidemic of associated cases among patients attending the outpatient clinic, which coincided with the beginning of the outbreak among the residents. It was not possible to identify the specific medical service that became the place where the outbreak started, although most of the cases appeared in the outpatient services. The incubation periods among the cases do not agree with the resident rotation periods.

In an epidemic outbreak, it is important to identify the etiological agent of infection. Some cellular lines are susceptible to infection by adenoviruses. Nevertheless, not all Ad types can be isolated, and the isolation technique requires at least 15 days [12]. In nine of the studied cases, the virus could be isolated using the VERO cell line. Because the virus in the index case could not be isolated, it was not possible to recognize the outbreak until the last four months of the year 2006. Starting with the second case of outbreak, primers were designed that allowed amplification of a region that identifies 14 Ad types [15]. Sequencing indicated that the agent responsible for the outbreak was Ad8. The delay in recognizing the etiological agent could have represented a risk factor in the virus dissemination among the medical personnel. Although the attack rate for hemorrhagic conjunctivitis was very high (24.39/100) and the period in which cases occurred was quite a long duration, no associated cases were reported among the relatives of the medical residents. Identification of genomic variants of the virus is therefore very important to assure that we were dealing with the same genotype.

The proposed outbreak containment scheme allowed effective measures to be taken, although at the time that these were implemented, only the etiological agent of the second case of outbreak was known.

An outbreak within our institute caused by Ad8 from a disseminated source was characterized by molecular and epidemiological studies. Because the transmission of this agent is through direct contact, it is reasonable to believe that transmission occurred due to relaxation of hygiene measures during the handling of patients. Therefore, we can be certain that the conjunctivitis among the residents was not random. It was originated by faulty basic hygiene measures.

Based on clinical manifestations and investigation of the literature, we can infer that the transmission was through direct, person-to-person contact. This means of transmission was corroborated by the successful containment of the conjunctivitis outbreak by blocking the chain of transmission between the personnel and patients. In summary, we detected an epidemic of hemorrhagic conjunctivitis among the patients cared for at our ophthalmology institute. Analysis of these cases revealed an unexpected increase, characteristic of a common source, which suggests that the common source was the institute itself.
